# SOCIAL NETWORK STRUCTURE AND THE WELL-BEING OF FAMILY CAREGIVERS TO OLDER ADULTS

**DOI:** 10.1093/geroni/igad104.0494

**Published:** 2023-12-21

**Authors:** Esther Friedman, James Murphy, David Kennedy

**Affiliations:** University of Michigan, Ann Arbor, Michigan, United States; RAND, Santa Monica, California, United States; RAND, Santa Monica, California, United States

## Abstract

Personal networks provide social support to both older adults and the family members who care for them, yet we know very little about the different structures of caregiver networks and which are most protective of caregiver well-being. Using a new nationally representative survey of 2,176 individuals who report providing care to a family member or friend age 50 or older with a health condition during the height of the Covid-19 pandemic and give detailed information on their personal networks, we examine caregivers’ networks in terms of size, density, proportion kin, and how care is allocated among network members. We also examine the implications of network structure for caregiver well-being including anxiety, depression, and hopefulness. We use latent profile analysis to identify three classes of caregiving networks: (1) Large, sparse networks with relatively few kin and only some people assisting with caregiving tasks for care recipients; (2) Moderately-sized, somewhat dense, kin-based networks; (3) Small, very dense kin networks where caregiving is shared across network members. Caregivers who are male, highly-educated, and providing care for someone with dementia are disproportionately in Class 1. Being in Class 3 is significantly associated with lower caregiver anxiety compared to being in the other two groups, even after controlling for sociodemographic factors, but not with depression and hopefulness. This work has implications for the optimal network structure for facilitating the well-being of family caregivers, and provides a baseline for examining network change and the implications thereof as the impact of the pandemic wanes.

